# Development of a SNP panel dedicated to parentage assignment in French sheep populations

**DOI:** 10.1186/s12863-017-0518-2

**Published:** 2017-05-26

**Authors:** F. Tortereau, C. R. Moreno, G. Tosser-Klopp, B. Servin, J. Raoul

**Affiliations:** 1 GenPhySE, INRA, INPT, INP-ENVT, Université de Toulouse, Castanet-Tolosan, France; 2 Institut de l’Elevage, DGEP, Chemin de Borde Rouge, F-31326 Castanet-Tolosan, France

**Keywords:** Sheep, Parentage assignment, SNP, Breeding programs

## Abstract

**Background:**

The efficiency of breeding programs partly relies on the accuracy of the estimated breeding values which decreases when pedigrees are incomplete. Two reproduction techniques are mainly used by sheep breeders to identify the sires of lambs: animal insemination and natural matings with a single ram per group of ewes. Both methods have major drawbacks, notably time-consuming tasks for breeders, and are thus used at varying levels in breeding programs. As a consequence, the percentage of known sires can be very low in some breeds and results in less accurate estimated breeding values.

**Results:**

In order to address this issue and offer an alternative strategy for obtaining parentage information, we designed a set of 249 SNPs for parentage assignment in French sheep breeds and tested its efficiency in one breed. The set was derived from the 54 K SNP chip that was used to genotype the thirty main French sheep populations. Only SNPs in Hardy-Weinberg equilibrium, displaying the highest Minor Allele Frequency across all the thirty populations and not associated with Mendelian errors in verified family trios were selected. The panel of 249 SNPs was successfully used in an on-farm test in the BMC breed and resulted in more than 95% of lambs being assigned to a unique sire.

**Conclusion:**

In this study we developed a SNP panel for assignment that achieved good results in the on-farm testing. We also raised some conditions for optimal use of this panel: at least 180 SNPs should be used and a minute preparation of the list of candidate sires. Our panel also displays high levels of MAF in the SheepHapMap breeds, particularly in the South West European breeds.

**Electronic supplementary material:**

The online version of this article (doi:10.1186/s12863-017-0518-2) contains supplementary material, which is available to authorized users.

## Background

Pedigree information is essential for accurate genetic evaluation. However, in French sheep populations the rate of known sires can vary widely, from a few percent in hardy breeds reared in high mountain areas up to 100% in specialized meat and dairy breeds. The lack of complete pedigrees and misidentification of sires affect the accuracy of genetic evaluation and consequently the efficiency of breeding programs [2, 9, 16, 29]. By increasing the percentage of known sires, the genetic gain of a breeding scheme is increased [24]. To identify the sire of a lamb, matings have to be controlled using Animal Insemination (AI) or natural matings with a single ram per group of ewes. The development of AI in sheep is unequal among breeds, particularly because (i) if fresh semen is to be used as recommended [21] the geographical area in which it can be applied is limited, (ii) the cost can be prohibitive to breeders compared with the economic value of a ram, and (iii) fertility is often lower than in natural mating conditions. When AI is not used, paternity is assessed through single-sire natural matings by managing several groups of ewes. This is very time-consuming for breeders especially for large flocks. This method is almost impossible to set up if the sheep are not confined and graze large pasture areas. For all these reasons, the number of ewes belonging to breeding program nucleus remains limited (mainly when paternity records are required by the breeding society) and the level of known paternities cannot be increased solely by improving the management of reproduction.

With the development of genotyping technologies, single nucleotide polymorphisms (SNPs) can be used to directly assign new born lambs to their true sire. In cattle, such parentage assignment has already been developed [10, 12, 33]. In sheep, SNPs dedicated to parentage testing have been selected in a set of international breeds [13]. At the International level, SNP parentage panels have already been set up in Australia [4], New Zealand [6] and North America [13].

In the study of Heaton et al. [13], based on the SheepHapMap design [19], only two French populations of the same breed (meat and dairy Lacaune) were included. However, in France there exist twenty-two breeding programs for twenty-one meat sheep breeds and six breeding programs for five dairy sheep breeds. Because of this large diversity of sheep breeds [20, 25] and because only two of them were included in the SheepHapMap design, we developed a specific SNP panel for parentage assignment that can be used in most French breeds. In this paper, we discuss our strategy for SNP selection, the results of the first use of the panel for parentage assignment and insights into its potential applicability for other populations across the world included in the SheepHapMap project.

## Methods

### Samples and genotyping

Thirty French sheep populations were sampled. These populations were selected among the 56 French breeds (http://www.racesdefrance.fr) because they register pedigrees as part of their own breeding program and are therefore most likely to be the main users of an assignment tool. Twenty-seven out of these thirty selected populations were genotyped with the Illumina Ovine Infinium® HD SNP BeadChip (603,350 callable SNPs– Illumina©). The three remaining populations had already been genotyped with the Illumina OvineSNP50 BeadChip 54 K SNP chip (54,241 SNP -Illumina ©) (Table 1). For each population, approximately thirty of the most unrelated and most representative males of the current genetic variability existing in their breed were selected. They were all selected in central testing station, all born after 2000 in different flocks, most of them without common ancestors in previous 3 generations and with on average 40 daughters (from 2 to 450 in meat breeds and from 10 to 1200 in dairy breeds) with production records (dairy yield or prolificacy).

**Table 1:** Characteristics of the genotyped breeds. The first two columns correspond respectively the short and full name of the breeds and the third column indicates the breed aptitude

Population	Population name	Meat/Milk	SNP genotyping^a^	Nb. of genotyped individuals
OIF	Ile de France	Meat	HD	27
CHL	Mouton Charollais	Meat	HD	27
RAVA	Rava	Meat	HD	25
CHM	Charmoise	Meat	HD	30
BCF	Berrichon du Cher	Meat	HD	30
LIM	Limousine	Meat	HD	25
LAC-OM	Lacaune-Ovitest	Meat	HD	24
LAC-GID	Lacaune-GID	Meat	HD	15
LAC-OL	Lacaune-Ovitest	Milk	HD	17
LAC-CONF	Lacaune-Confederation	Milk	HD	23
TEX	Texel	Meat	HD	26
SUF	Suffolk	Meat	HD	28
MERA	Merinos d’Arles	Meat	HD	24
PAS	PreAlpes du Sud	Meat	HD	26
NDV	Noire du Velay	Meat	HD	26
BMC	Blanche du Massif Central	Meat	HD	26
BB	Basco-Bearnaise	Milk	54 K	30
CDL	Causse du Lot	Meat	HD	29
ROMV	Romanov	Meat	HD	17
GRI	Grivette	Meat	54 K	30
VDN	Mouton Vendeen	Meat	HD	29
RO	Rouge de I’Ouest	Meat	HD	29
TAR	Tarasconnaise	Meat	HD	25
COR	Corse	Milk	HD	29
MOUR	Mourerous	Meat	HD	24
MTN	Manech Tete Noire	Milk	54 K	29
MTR	Manech Tete Rousse	Milk	HD	26
ROUS	Roussin	Meat	HD	29
ROME	Romane	Meat	HD	23
MBB	Martinique Black Belly	Meat	HD	23

^a^:"HD” stands for the Ovine Infinium® HD SNP BeadChip (603,350 callable SNPs- Illumina©) and “54 K” stands for the Illumina OvineSNP50 BeadChip 54 K SNP chip (54,241 SNP -Illumina ©)

Additional individuals originating from experimental flocks with reliable pedigrees were used to identify markers with high rates of Mendelian transmissions errors: a Romane x Martinik Black Belly backcross [27], a Romane pedigree [11] and a Lacaune pedigree [26]. From these flocks, we used the genotypes of 413 trios “lamb-dam-sire” (413 lambs born from 245 dams and 32 rams).

### Parentage SNP panel selection

Two different genotyping chips were used and only the 42,230 SNPs present on both chips were initially pre-selected for evaluation. Then, four selection steps were applied successively:

1. biallelic SNPs with a known location on one of the 26 autosomes (on assembly Oar_v3.1, http://www.livestockgenomics.csiro.au/sheep/oar3.1.php) and with a genotype calling frequency greater than 0.99 were retained,
2. the minor allele frequency (MAF) was calculated for each SNP in each of the thirty populations and SNPs with the following features were selected: MAF greater than 0.30 in at least 20 populations, between 0.20 and 0.30 in at most 10 populations, between 0.10 and 0.20 in at most one population, and greater than 0.10 in all the 30 populations,
3. the Hardy-Weinberg Equilibrium was tested within each of the thirty populations and SNPs that were not at equilibrium in at least one population were discarded,
4. individuals from experimental flocks with verified parentage were used to check for Mendelian inconsistencies and only SNPs devoid of errors were retained.

Finally, to obtain a final subset of about 250 not redundant SNPs, we selected SNPs with a MAF greater than 0.30 in the largest number of populations per 10 Mb-window. Linkage disequilibrium (r^2^) between pairs of SNPs of the final set was calculated with PLINK [22].

### Parentage SNP panel efficiency

To assess the assignment efficiency of the SNP panel, we considered the following criteria:

- *The exclusion probability (PE)* (probability to exclude one (PE1) or two (PE2) randomly sampled parent(s) from the parentage of an individual which is truly unrelated to them) was calculated for the final panel for each of the 30 populations using the usual formulae [28].
- *The probability of identity (PI)* (probability that two randomly selected individuals in a population have identical genotypes) calculated as:

$PI={\prod}_{i=1}^{Nsnp}\left({freqAA}^2+{freqAB}^2+{freqBB}^2\right)$, with freqAA, freqAB, freqBB being the relative genotype frequencies of AA, AB and BB individuals respectively, for a biallelic SNP with alleles A and B.

These criteria highly depend on the number of SNPs. In order to compare on an equal level the efficiency of our panel to other existing SNP panels, we randomly sampled 96, 150 and 200 SNPs of the final selected subset. One thousand samplings were performed per density and for each sampling we calculated PE1, PE2, PI and the mean MAF for each of the 30 populations studied.

### Testing the performance of the SNP panel for assignment (animals, genotyping technology and assignment methodology)

The SNP set was tested using an on-farm design for BMC (*Blanche du Massif Central*) sheep. Blood samples were collected from 509 individuals: 281 lambs, 105 sires and 123 dams. The lambs were produced by monospermic AI (the semen of a single ram was used) so their sire was assumed as known based on the breeder’s records. Genotyping was performed using Sequenom technology [8]. Parentage assignment of lambs was based on its parents’ likelihood contributions for each marker which were obtained using an in-house script based on the method developed by Boichard et al. [5]. A likelihood ratio was calculated between the likelihood estimated for a given parent and the likelihood obtained with a virtual parent with the allelic frequencies estimated in the population. The dam was confirmed when the likelihood ratio was positive and when there were less than 10 Mendelian incompatibilities with the lamb. Lambs with unconfirmed dams were removed from the following analyses. In order to select the most likely sire, the posterior parentage probabilities were calculated for each candidate sire. The posterior parentage probabilities of each candidate sire were computed from their respective likelihoods, assuming all candidate sires were a priori equally likely to be the true sire. A candidate sire was finally assigned as true sire if (i) the likelihood ratio was positive, (ii) the posterior probability was greater than 0.99 and (iii) the number of mismatches with the offspring was lower than 10. These two steps were followed for all the 281 lambs, and the results of paternal assignment testing were used to calculate the performance of the assignment procedure (i.e. SNPs genotyping and paternal assignment method). The robustness of our panel was finally tested: four true sires were removed from the list of candidate sires and assignment results of their lambs were analyzed. The four removed sires were chosen because other related sires (their sire and/or half-sibs and/or offspring) were included in the list of candidate sires.

### Comparison of the performances of the French SNP panel and other international panels in various French and international breeds

The SNPs included in the French panel were selected based on the genetic diversity of French sheep breeds. The MAFs of the panel’s SNPs were estimated in the different international populations involved in the SheepHapMap project [19].

We also compared the MAF, PI, PE1 and PE2 obtained with two additional SNP parentage panels developed for New-Zealander [6] and North American breeds [13] in the thirty French populations. These two panels respectively include 163 (North America) and 98 (New Zealand) SNPs, with 55 SNPs in common between the two sets.

## Results

### Parentage SNP selection

In the following analyses, only individuals with a genotyping callrate greater than 0.95 were retained. This reduced the final number of useful genotyped individuals to 771 (Table 1), with 15 to 30 individuals per breed.

To ensure the applicability of the final SNP panel was optimal, we considered only the 42,230 SNPs in common between the high and medium density SNP chips as candidates for inclusion in the panel. In order to reduce as much as possible the genotyping cost related to the use of the final parentage panel, while maintaining its efficiency for assignment, we decided to include 200–300 SNPs.

On average the genotype calling frequency was 0.985, and we initially selected the 32,692 SNPs (~80%) that had a frequency greater than 0.99.

**Fig. 1:**
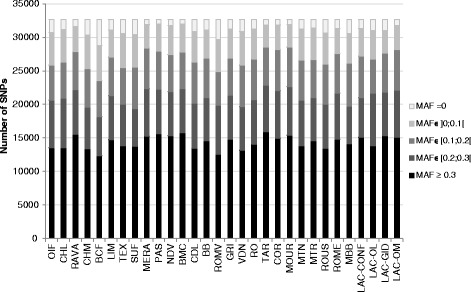
Number of SNPs (among 32,692 SNPs after routine editing including a call-freq higher than 0.99) per MAF class for the 30 populations

**Fig. 2:**
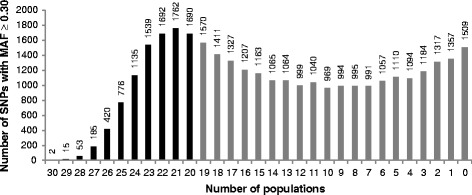
Number of SNPs with MAF ≥ 0.3 in a given number of populations. For example, there are 1135 SNPs having a MAF ≥ 0.3 in exactly 24 populations. Black and grey bars correspond to SNPs that were selected and discarded respectively for the next selection steps

For each of the 30 populations, the MAF distribution of these 32,692 SNPs was calculated (Fig. 1). In each population at least 12,300 SNPs have a MAF > 0.30. However, MAF-based SNP selection had to be performed at the same time for the 30 populations studied. Because only 2 SNPs have a MAF > 0.30 in all the populations (Fig. 2), additional selection criteria were applied as previously described. A total of 9269 SNPs had a MAF > 0.30 in at least 20 populations (Fig. 2), but only 1929 SNPs met all the criteria.

Among these 1929 selected SNPs, 453 departed from the Hardy-Weinberg equilibrium (*p* < 0.01) in at least one of the thirty populations. This reduced the number of candidate SNPs to 1476. Among the 453 discarded SNPs, two were not at equilibrium in three populations, 55 were not at equilibrium in two populations and 396 were not at equilibrium in just one population.

The analysis of 262 lambs of a backcross design with genotypes for all the 1476 candidate SNPs, as well as their two validated parents, highlighted 44 SNPs with at least one Mendelian transmission error.

**Fig. 3:**
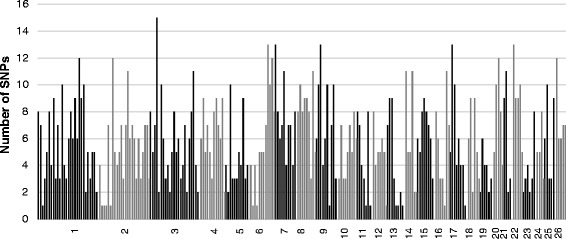
Distribution of the 1432 SNPs over the 26 autosomes. Each bar represents the number of SNPs per a 10 Mb window

**Fig. 4:**
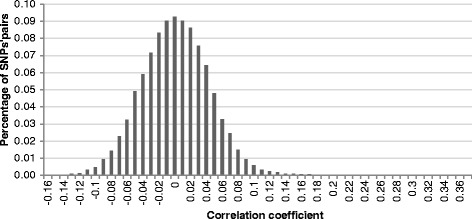
Distribution of the genotypic correlations calculated between pairs of SNPs (SNPs from the 249 final panel)

After these four selection steps, 1432 SNPs were therefore identified as good candidates for the parentage assignment panel across the thirty considered populations. The average MAF per SNP was 0.37 (ranging from 0.33 to 0.42); the lowest average MAF was observed in the BCF (*Berrichon du Cher*) population (0.34) and the highest in the PAS (*PréAlpes du Sud*) population (0.39). These 1432 SNPs were unequally distributed over the genome (Fig. 3), with 1 to 15 SNPs per 10 Mb window. A final set of 249 SNPs was obtained by selecting one SNP per 10 Mb window. On average, there was no correlation (0.0015 ± 0.0435) between the genotypes of two SNPs sampled from the panel of 249 SNPs (Fig. 4), and linkage disequilibrium was estimated at 0.0018 ± 0.0025.

**Fig. 5:**
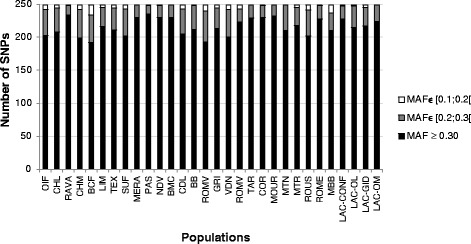
Number of SNPs (among the 249 selected ones) per MAF class for the 30 populations

The 249 selected SNPs had an average MAF of 0.39, ranging from 0.33 to 0.42 (Additional file 1: Table S1). At the population level, the lowest average MAF was obtained in the BCF population (0.360) and the highest in the MOUR (*Mourerous*) population (0.408) (Table 2). The BCF population was the population with the less favorable situation in terms of MAF distribution with 192 SNPs with a MAF higher than 0.30, 42 SNPs with a MAF between 0.20 and 0.30 and 15 SNPs with a MAF between 0.10 and 0.20 (Fig. 5). The probability (PI) that two randomly selected individuals having identical genotypes with this panel of 249 SNPs was very low: it reached its lowest value in the BMC population (6.3 × 10^−100^) and its highest value in the GRI (*Grivette*) population (3.91 × 10^−92^). The exclusion probabilities of either one or the two randomly selected parent(s) (PE1 and PE2 respectively) were close to 1 in all 30 populations (Table 2).

**Table 2:** Major statistics for three parentage panels (French, New Zealand, North America) on the 30 French populations: MAF, PI (Probability of identity), PE1 and PE2 (exclusion probabilities considering the exclusion of one or the two parents respectively)

French panel (249 SNPs)					New Zealander panel (98 SNPs)				North American panel (163 SNPs)			
	MAF	PI	1-PE1	1-PE2	MAF	PI	1-PE1	1-PE2	MAF	PI	1-PE1	1-PE2
OIF	0.381	7.90E-96	7.24E-13	2.63E-34	0.310	1.00E-30	3.56E-04	2.26E-11	0.337	1.11E-52	5.70E-07	2.97E-19
CHL	0.387	9.95E-97	4.78E-13	1.68E-34	0.339	2.15E-32	1.65E-04	6.10E-12	0.345	4.44E-53	4.40E-07	1.94E-19
RAVA	0.398	6.71E-98	1.98E-13	6.33E-35	0.351	2.05E-32	1.21E-04	4.06E-12	0.367	1.34E-53	1.94E-07	8.77E-20
CHM	0.367	1.95E-94	1.81E-12	6.85E-34	0.303	6.79E-30	4.62E-04	3.33E-11	0.319	1.90E-51	1.33E-06	1.12E-18
BCF	0.360	6.67E-93	3.44E-12	1.83E-33	0.264	1.41E-26	1.32E-03	5.73E-10	0.300	2.65E-47	3.44E-06	2.40E-17
LIM	0.384	2.48E-95	5.97E-13	2.03E-34	0.331	1.47E-31	2.28E-04	1.29E-11	0.346	4.24E-52	4.70E-07	2.94E-19
TEX	0.388	2.76E-96	4.36E-13	1.55E-34	0.321	1.37E-31	2.83E-04	1.60E-11	0.339	3.56E-53	5.94E-07	3.64E-19
SUF	0.375	2.87E-95	9.05E-13	2.82E-34	0.322	9.01E-32	2.54E-04	1.00E-11	0.333	7.16E-53	6.84E-07	5.19E-19
MERA	0.408	3.39E-98	1.15E-13	3.76E-35	0.344	8.23E-33	1.28E-04	3.82E-12	0.372	1.75E-55	1.16E-07	3.82E-20
PAS	0.408	7.65E-97	1.15E-13	3.81E-35	0.354	4.13E-33	9.45E-05	2.58E-12	0.388	3.25E-56	6.33E-08	2.05E-20
NDV	0.394	8.50E-99	2.43E-13	7.14E-35	0.352	8.56E-33	1.12E-04	3.56E-12	0.368	6.28E-56	1.53E-07	4.79E-20
BMC	0.405	6.33E-100	1.26E-13	3.98E-35	0.363	1.86E-33	8.52E-05	2.69E-12	0.394	6.32E-58	4.74E-08	1.50E-20
CDL	0.383	1.83E-94	6.05E-13	2.21E-34	0.326	5.55E-31	2.37E-04	1.35E-11	0.336	3.28E-52	6.25E-07	3.22E-19
BB	0.376	1.56E-95	8.53E-13	2.52E-34	0.318	1.92E-30	2.93E-04	1.73E-11	0.344	3.48E-52	4.45E-07	2.26E-19
ROMV	0.373	1.02E-93	1.42E-12	5.66E-34	0.309	2.44E-30	3.92E-04	1.62E-11	0.319	5.15E-51	1.46E-06	1.17E-18
GRI	0.379	3.91E-92	7.89E-13	2.75E-34	0.345	1.16E-31	1.46E-04	6.46E-12	0.356	1.80E-52	2.95E-07	1.69E-19
VDN	0.371	1.14E-94	1.30E-12	4.73E-34	0.322	7.05E-32	2.59E-04	1.30E-11	0.322	2.80E-51	1.25E-06	1.20E-18
RO	0.390	2.97E-98	3.53E-13	1.19E-34	0.323	6.54E-31	2.76E-04	1.38E-11	0.345	5.46E-53	4.36E-07	4.82E-19
TAR	0.388	3.72E-97	3.45E-13	1.00E-34	0.333	5.88E-32	1.96E-04	6.86E-12	0.369	2.03E-55	1.28E-07	3.97E-20
COR	0.401	5.39E-98	1.61E-13	5.14E-35	0.356	2.44E-32	1.16E-04	4.77E-12	0.359	5.43E-55	2.04E-07	7.24E-20
MOUR	0.408	4.67E-99	1.02E-13	3.25E-35	0.352	3.13E-33	1.12E-04	3.62E-12	0.377	1.69E-56	1.06E-07	3.43E-20
MTN	0.382	2.77E-96	5.48E-13	1.59E-34	0.323	4.61E-31	2.72E-04	1.71E-11	0.340	4.20E-53	6.34E-07	4.38E-19
MTR	0.389	3.97E-98	3.56E-13	1.19E-34	0.323	1.74E-31	2.32E-04	9.91E-12	0.342	1.63E-53	4.66E-07	1.88E-19
ROUS	0.374	1.53E-95	1.15E-12	4.53E-34	0.304	4.74E-30	4.57E-04	4.86E-11	0.340	3.10E-52	5.82E-07	5.32E-19
ROME	0.406	1.27E-98	1.13E-13	3.72E-35	0.323	1.32E-30	2.41E-04	1.26E-11	0.365	1.45E-53	1.69E-07	7.83E-20
MBB	0.370	1.21E-93	1.40E-12	5.33E-34	0.312	1.13E-30	3.49E-04	1.81E-11	0.336	1.81E-51	6.46E-07	4.42E-19
LAC-CONF	0.399	3.02E-96	2.00E-13	6.28E-35	0.349	3.02E-32	1.17E-04	5.58E-12	0.361	1.07E-54	1.89E-07	7.45E-20
LAC-OL	0.392	1.05E-94	2.95E-13	9.16E-35	0.332	6.06E-31	2.20E-04	1.16E-11	0.364	2.44E-53	2.05E-07	1.05E-19
LAC-GID	0.385	3.82E-95	5.46E-13	1.76E-34	0.329	3.48E-31	2.08E-04	8.85E-12	0.353	1.06E-52	3.22E-07	1.28E-19
LAC-OM	0.400	1.76E-97	1.85E-13	5.89E-35	0.370	6.05E-33	7.62E-05	2.67E-12	0.385	1.65E-55	7.40E-08	2.46E-20

When we randomly selected different SNP densities (96, 150 or 200SNPs) among the 249 selected ones, we observed no difference on the average MAFs. However, PE1 and PE2 increased with the number of SNPs whereas PI decreased at higher panel densities (Additional file 2: Table S2).

### Testing parentage assignment with the selected SNPs

The parentage assignment procedure was tested using Sequenom technology for genotyping. For economic reasons only four plexes were developed. For technologic reasons, these four plexes included 192 SNPs among the 249 previously selected SNPs and were used to genotype 509 individuals from the BMC breed. Based on genotyping quality, the number of useful SNPs decreased to 181.

Before performing sire assignment, we assessed the genetic link between the lambs and their declared dams (Table 3). Based on the likelihood and on the number of incompatibilities, 12 lambs were removed from subsequent analyses. The other 269 lambs were included in the sire assignment test. For the 174 lambs displaying a genotyped and validated dam, the sire assignment rate reached 97%. For the remaining 95 lambs with no genotyped dam, the sire assignment rate was 93.7%. If all lambs were considered together, whatever the genotyping status of their dam, the assignment rate reached 96%. For 233 lambs, the assigned sire matched the declared sire (90% agreement). For 25 lambs, the assigned sire did not match the declared sire. Among the 269 lambs, 258 were finally sire-assigned. For these 258 assigned lambs, the average likelihood ratio was 14.4 ± 3.6, ranging from 5.8 to 26.04, and there were on average 3.1 Mendelian incompatibilities (with a maximum of 8) between the sire and the lamb.

**Table 3:** Results of the paternal assignment test in the BMC breed

		Nb. lambs with genotyped and validated dam	Nb. lambs with ungenotyped dam	Nb. lambs with genotyped but unvalidated dam
Lambs with declared sire (*n* = 281)	Nb. lambs with confirmed sire	152	81	
	Nb. lambs with declared sire different from assigned sire	17	8	12
	Nb. lambs with no assigned sire	5	6	
	Total	174	95	12

We then tested the robustness of our SNP panel. We proposed to assign 82 lambs to a list of 101 sires from which their four true sires (assigned in the previous step) had been removed. As a consequence, we expected that no paternal relationship would be found. Among these 82 lambs, 64 had a confirmed dam and the dam was unknown for the 18 remaining lambs. The list of candidate sires still included relatives of the 4 discarded sires (i.e. their sire and/or half-sibs and/or progeny). All the 64 lambs with a confirmed dam were not assigned. However, six out of the 18 lambs with unknown dam were assigned to a sire. These falsely assigned sires were paternal half-sibs of the six lambs.

### MAFs for French parentage SNPs in worldwide breeds

**Fig. 6:**
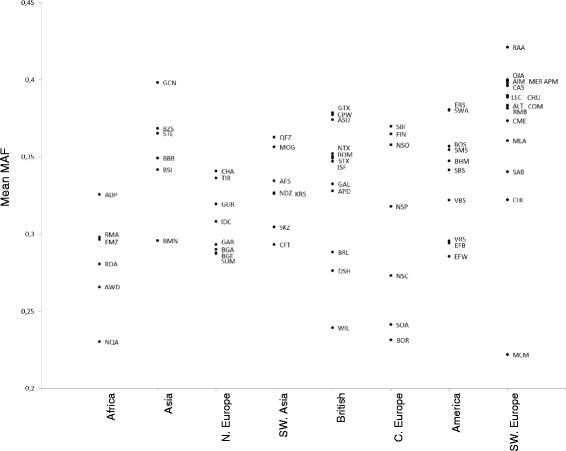
Mean MAF per population in the eight breed group included in the Sheep HapMap project [19]. MAF were estimated with the 249 SNPs of the French parentage panel

The 249 SNPs selected for French breeds were analyzed in the set of worldwide breeds described in [19]. The populations from South West Europe showed the highest mean MAF (0.38), particularly with Spanish breeds which displayed a MAF around 0.4. On the opposite, African populations showed the lowest mean MAF (0.28) (Fig. 6).

### MAFs for international parentage SNP panels in French breeds

Two parentage SNP panels (from New Zealand and Northern America) selected on different international breeds were analyzed in the 30 French populations of this study. Among the 1432 SNPs that fulfilled all our criteria (before genomic distribution selection), 6 and 25 SNPs were also included in the New Zealander and North American panels respectively. Finally, two and eight SNPs from the New Zealander and North American panels respectively were also included in the French panel. The MAFs estimated for these two international panels in the thirty French populations were 0.33 for the New Zealander panel and 0.35 for the North American panel. These values are to be compared with the average MAF of 0.39 for the French panel. As shown in Table 2, the mean MAF observed per population is highest for the French panel, and mean MAFs are higher for the North American panel compared with the New Zealander panel. If we consider PI, PE1, PE2 values, the North American panel displays a higher PE and a lower PI than the New Zealander panel, but neither of them reach the results obtained with the French panel (Table 2). When we randomly sampled 96 or 150 SNPs to use densities close to those of the New Zealander and North American panels respectively, the results with the randomly sampled French sub-panels were better for all the 30 populations than with the two international panels. Indeed, PI was lower with 96 SNPs than with the New Zealander panel and both exclusion probabilities were higher with 96 SNPs. By randomly selecting 150 SNPs, we obtained lower PI and higher exclusion probabilities for all the 30 populations than with the panel from North America which contains 163 SNPs (Additional file 2: Table S2).

## Discussion

### Design of the SNP parentage panel

In this study, we report the development of a SNP panel dedicated to parentage assignment which is suitable for most of the French sheep breeds. Until now, parentage verification methods proposed to French sheep breeders have relied on the use of microsatellite markers. Microsatellite panels used for parentage verification have been tested for parentage assignment but were shown to lack in power for such analysis [23]. Whereas parentage verification can be used to validate or not a sire, parentage assignment enables breeders to identify the true sire amongst a list of candidate sires. Due to recent advances in molecular technology, SNPs are now of particular interest because their analysis can be entirely automated and they are gradually becoming the most used markers for parentage analysis [1]. The development of a dedicated panel for parentage assignment would provide breeders with the opportunity of making mating management easier while improving the known paternity rate. We opted for SNPs as molecular markers in order to develop a panel that can be genotyped automatically and at a more reasonable cost than microsatellites. The number of SNPs needed to assign individuals to their parents has been recently estimated in cattle and sheep: using the exclusion probability, Strucken et al. [31] recommended to use at least 200 markers and to increase this number to solve conflicting results. Our choice of a panel comprising 200–300 SNPs was rather large compared to other assignment panels available for both species which include 80 to 200 SNPs [6, 13, 15], but allowed us to develop an assignment panel suitable for all French sheep breeds with established breeding programs. In cattle, it has been shown that the full ISAG (200 SNPs) parentage panel is efficient in a wide variety of breeds, but when the number of SNPs must be decreased (for technical and/or economic reasons), population-specific panels are more efficient [30]. Based on simulations, Boichard et al. advised to use at least 175 SNPs if the targeted populations display “unfavorable” conditions such as non-genotyped dams, a partly genotyped set of sires and/or highly-related candidate parents [5]. This is slightly higher than the range of 100–150 SNPs proposed by Hill et al. if the list of potential parents includes highly-related individuals (such as full sibs, sires-offspring) [14]. In French sheep populations we are close to such “unfavorable conditions” because many sets of candidate sires include related males (for example sons-sires, half-full sibs). This was for example the case when testing the BMC population for which the list of candidate sires included some parent-offspring pairs. Due to these particular population structures, we decided to select 200–300 SNPs in order to meet the recommendations provided by Boichard et al. [5]. After filtering, we identified 1432 candidate SNPs covering all the genome. We finally selected 249 evenly-spaced SNPs (one SNP per 10 Mb window). There was no redundancy among these 249 SNPs as indicated by the low level of linkage disequilibrium. It should be noted that the 249 assignment SNPs were selected without preconceived ideas on which genotyping technology will finally be routinely used. We decided to retain all 249 SNPs knowing that, depending on the genotyping tool, the number of SNPs actually used could decrease. As mentioned before, a minimum number of at least 175 SNPs had to be genotyped in order to have high rates of sire assignment. Our on-farm validation of the panel indicates that the panel should include at least 180 SNPs given the number of false-positive results when the dam is not genotyped and the true sire is not among the candidate sires.

Concerning the maximum number of SNPs, the main criteria was the cost of the genotyping. From an economical point of view we could not afford to select more than 300 SNPs because the final panel will be used by breeders so the cost of its use must be as low as possible. Raoul et al. estimated that using parentage assignment to increase the pedigree information could be profitable for a cost per assignment close to 6–7 € [24].

To select assignment SNPs, we mainly focused on MAF analysis, i.e. the standard procedure for developing such parentage panels [6, 13], given that the number of SNPs needed for assignment decreases when the MAF increases [3]. In our study, a first step based on this criterion led to a major reduction in candidate SNPs (from 32,692 to 1929) given that we considered thirty populations at a time. When the selection of parentage SNPs relies on few populations, more stringent MAF thresholds can be applied, and additional selection criteria are needed to decrease the number of parentage SNPs [32].

In our study, we also calculated the exclusion and identity probabilities obtained for each French population for the panel of 249 SNPs. These probabilities are highly dependent on the number of SNPs [7, 17]. In order to compare our panel to the panels from North America and New Zealand, we randomly sampled 96, 150 and 200 SNPs from the 249 selected SNPs. With less SNPs than the panel dedicated to North American breeds, we obtained better results for the French breeds as regards to PE and PI. Our results confirm that a greater number of SNPs results in a decrease of PI and an increase of PE, but also reveal that when the number of SNPs must be decreased (for technical or economic reasons), PE and PI levels can be maintained by specifically selecting SNPs adapted to the populations to which the panel is designed.

To assign lambs to their sire, we used the likelihood methodology which accounts for genotyping errors and allow missing genotypes. Other methodologies exist which usually only rely on exclusion [18]. However, likelihood approaches achieve better results than exclusion approaches as illustrated by Boichard et al. particularly when there are genotyping errors [5]. To perform assignment, we removed all lambs with incompatible dams based on genotype information because we could not rule out the possibility that the blood sample had been mislabeled. We used 3 criteria to assign a lamb to a sire: the likelihood ratio, the posterior probability and the number of Mendelian incompatibilities between the lamb and the sire. The likelihood ratio was the first criteria, but it is not sufficient alone as a lamb could have a positive likelihood ratio with two or three sires. We added the posterior probability as a second criteria to retain the most likely sire, and it could return only one sire per lamb because of the threshold (0.99) we applied. For technical reasons, we allowed a fairly high maximum of Mendelian errors (10) with on average three Mendelian incompatibilities between a lamb and its assigned sire. With improved genotyping quality, a more stringent threshold could be applied (i.e. 5 incompatibilities).

### A French parentage panel dedicated to French breeds, but of interest for other European breeds

Various SNP parentage panels already exist for sheep of different international breeds. The panel presented by Clarke et al. was developed based on New Zealand breeds [6]. The panel proposed by Heaton et al. was developed based on the SheepHapMap database (where the only French breed represented is the Lacaune breed) and on a U.S sheep panel (Heaton et al. [13]). We proposed in this study to select SNPs based on the genetic diversity of French breeds and to compare the quality of the different panels for French breeds. If we consider MAF as one of the criteria to evaluate the quality of parentage panels, the French panel performs best for the French breeds, followed by the North American panel and finally the New Zealander panel. It should be noted that even if the panel developed for New Zealand breeds returns the lowest MAF for almost all the French breeds, these MAF are still higher than 0.3 (Table 2). However, if we focus on specific French breeds (for example BCF and ROME), the difference in MAF between the French panel and the other panel is around 0.10. Regarding the Lacaune breed (included in the SheepHapMap project), the French panel performs better than the two other panels in terms of mean MAF (Table 2). Similarly, better exclusion and identity probabilities are achieved with the SNPs of the French panel, even when the number of SNPs is decreased to reach a density close to that of the two international panels.

The French parentage panel was tested on-farm with individuals from the BMC breed. We obtained very encouraging results with 94% of individuals being assigned when the dam was not genotyped and up to 97% when the dams were also genotyped. However, with this on-farm design, we cannot be absolutely sure that all the candidate sires were sampled and genotyped, so it is likely that the true sires of some of the unassigned lambs were not in the list of candidate sires. By way of comparison, in commercial flocks where all the lambs and sires were sampled, on average 93% of lambs were sire-assigned with the New Zealander panel in a situation where only sire genotypes were considered [6], which is similar to the performance of our assignment procedure (SNPs and algorithm).

Even if our panel performed well for the BMC breed, significant emphasis should be put on the need for minute preparation of the list of candidate sires. We show in this study that if the true sire is not in the list but that some of its relatives are, false-positive assignments can be observed when dams are not genotyped.

At the European level, no SNP parentage panel has been published before the panel we propose here. Based on the MAF criteria, we believe our panel should perform well in breeds belonging to the following SheepHapMap subgroups: South West Europe (excepted Mac Arthur Merino population which is inbred), Italy, and to a less extent Central Europe, part of the Northern Europe sub-group, and America and South West Asia. For example, if we focus on the results obtained with the Spanish breeds, observed MAF are of the same order as most of the French breeds (approximately 0.4) (Fig. 6).

## Conclusion

In this study, we designed a SNP panel that will enable accurate parentage assignment in most of the French sheep breeds. This panel was established by genotyping approximately 30 individuals from 27 and 3 populations genotyped respectively with the 600 K and 54 K SNP chips.

The selected 249 SNPs were successfully tested for parentage assignment in the BMC breed with a minimum assignment rate of 94%. Even if very encouraging results were obtained in terms of paternity assignment rates, this study highlights a major condition to be met for the successful use of this new tool: when dams cannot be genotyped, the list of putative sires must be as complete as possible in order to prevent the risk of miss-assignment to a relative of the true sire.

This panel is currently being used in some French breeds. With an increasing number of assigned animals we will be able to assess on real datasets the benefits in terms of genetic evaluations, such as an improved accuracy of breeding values and connections between flocks, and in terms of pedigree-based genetic variability indicators.

## Additional files

Additional file 1: Table S1. List of the 249 SNPs selected for Parentage assignment in French breeds. For each SNP, the genomic position is given in reference to the oar_v3.1 assembly, and the MAF is the average over the 30 French breeds included in the analysis. (XLSX 23 kb)

Additional file 2: Table S2. Results (MAF, PE1, PE2 and PI) obtained after random sampling of SNPs from the 249 SNPs selected for parentage assignment in French sheep breeds. MAF, PE1, PE2 and PI statistics were obtained after 1000 random sampling of 96, 150 and 200 SNPs. PE1 and PE2 give the probability of exclusion of one or both parents respectively, and PI gives the probability of identity. (XLSM 33 kb)
